# Nursing Project Management to Reduce the Operating Room Infection

**Published:** 2017-02

**Authors:** Yuanyuan CHEN, Xiaodao HAN, Yongjie XU, Weihua LI

**Affiliations:** 1. Dept. of Nursing, Qilu Hospital of Shandong University, Jinan, Shandong, PR China; 2. Dept. of Gynecologic Oncology, Qingdao Central Hospital, Qingdao, PR China; 3. Hekou District People’s Hospital of Dongying City, Shandong, PR China; 4. Operating Room, Qilu Hospital of Shandong University, Jinan, Shandong, PR China

**Keywords:** Project management, Operating room infection, Nursing

## Abstract

**Background:**

Nursing project management is widely used in different aspects of the society. However, whether the nursing project management can control the infections in the operation room (OR) is rarely reported. We evaluated the outcomes of surgical patients after implementing a nursing project management program to provide new scientific ways to manage the OR infections.

**Methods:**

Overall, 382 patients, who underwent surgical treatment in Qilu Hospital of Shandong University, Shandong, China from May 2015 to January 2016, were enrolled as observation group. Besides, 347 cases were selected as control group. Patients in the observation group were treated with the nursing project management plan, while patients in the control group were treated with the routine operation-room nursing measures. The infection control rates in the OR, and the patient satisfaction with the nursing team postoperatively were both compared between the two groups of patients.

**Results:**

The OR air, the surgical and personnel’s hands surfaces were sampled for colony forming units, and all were found to be significantly of better quality (indicated by less colony forming units) in the observation group (P<0.001). In addition, there were 3 cases (0.79%) of post-operation infections in the observation group, while 12 cases (3.46%) occurred in the control group. The overall infection rate of the observation group was significantly lower than that of the control group (P = 0.011); and the satisfaction of patients with the nursing team in the observation group was significantly higher than that of the patients in the control group (*P* = 0.001).

**Conclusion:**

It is worth popularizing and applying a good nursing project management plan for surgical patients in hospitals.

## Introduction

Hospital acquired infections occur frequently in the gastrointestinal, respiratory and urinary tracts, and in surgical incision sites (which account for approximately 15% of all nosocomial infections) (1). Therefore, hospital operation room infection management is a very important aspect of the nosocomial infection, prevention and control program of a hospital, and it is an important part of the quality management in the operation room (2). The hospital operation room management unquestionably influences the rate of surgical incision infections, and a good nursing management program successfully reduces the hospital acquired infection rate (3).

Previous studies have focused on OP- aseptic management, equipment disinfection (4, 5), medical surgical principles and surgical operation process managements. These management plans are not systematic and have no support of evidence-based medicine. They do not follow the requirements to improve continuously the management quality, and cannot meet the demands of the development of new techniques, changes in patients’ situation and mobility of the medical staff. The nursing management team applies systematic theory and methods in order to plan, organize, coordinate, control, communicate an approach and motivate the medical personnel to achieve the common goal of infection prevention (4–5).

The nursing project management plan is characterized by individuality, periodicity and improvability. The individuality is targeted to analyze and solve a particular of a group of similar cases. The periodicity is to sum up and review the management work on regular intervals of time (no longer than 1 year). The improvability is to develop more scientific and effective management strategies to adapt to the ever-changing environment and evolving needs. The nursing project management has wide and valuable applications in the fields of economics, business and corporate development, social and public health. It is also applied for the development of hospitals and the management of departments of the hospitals. For example, it can shorten the average length of average hospital stay and reduce medical disputes.

Our hospital has applied one such management plans and the results are described in here.

## Materials and Methods

### Samples

Overall, 382 patients, who underwent surgical treatment in Qilu Hospital of Shandong University, China were continuously selected for the observation group from May 2015 to January 2016.

The inclusion criteria were: 1) The surgeries were noninfectious; 2) The surgeries followed the aseptic principles; 3) The patients had no antibiotic resistance; 4) The testing procedures were followed. The exclusion criteria were: 1) The patients had severe underlying diseases, such as heart, liver, lung, kidney, brain and other organ dysfunctions, autoimmune diseases, organ transplantations; or they needed glucocorticoids and cytotoxic drugs; 2) The patients had more than 2 surgeries in the past 1 year.

From those 171 were male and 211 were female; their ages ranged from 12 to 74 yr (43.8±13.5 years on average); orthopedic surgery was conducted for 196 cases, general surgery for 137 cases, and gynecologic surgery for 48 cases.

In parallel, 347 patients between January 2014 and May 2015 under the same conditions and during the same period were continuously selected as the control group. From them 162 were male and 185 were female; their ages ranged from 14 to 78 yr (45.3±15.0 yr on average); orthopedic surgery was conducted for 185 cases, general surgery for 119, and gynecologic surgery for 43. The differences in the general information between the two groups of patients had no statistical significance (*P*>0.05).

This study obtained the approval from the Ethics Committee of our hospital. The informed consents were taken from patients.

### Research methods

The control group was treated using routine surgical nursing measures, while the observation group was treated with measures of a designed surgical room nursing management project. The specifics were the following:

### Establishment of a project management team

The head nurse carried out the overall coordination and management planed a surgical project for each patient and oversaw the preoperative, intraoperative and postoperative nursing situation. A specially designated person monitored and recorded any relevant findings (6).

### Preoperative management

Nursing intervention directed at patients: patients were informed that sufficient nutrition was needed before surgery to strengthen bodies’ immune ability and anti-infection ability, and special care was applied when performing personal hygiene work. Nursing surgery-related intervention (7): it was ensured that surgical items and instruments completely met the sterilization requirements. The specially assigned person inspected and labeled the sterilized surgical instruments and items to prevent misplacements. Whenever the sterile condition of materials was unidentified, the materials were removed, changed or replaced immediately Items including surgical gowns, clean cloths, slippers, mops, and others were disinfected as requested. Infectious surgeries were properly identified on the surgical drape to help doctors plan accordingly, to prevent the occurrence of cross infections (8).

### Intraoperative nursing

Nursing anesthesia intervention: For general anesthesia intubation, disposable filters and oxygen masks were employed in order to decrease the infection rate during anesthesia, and the frequency of disinfection of screwed pipes was increased, since contamination of the laryngoscope has been reported as an important factor leading to surgical room infection (9). Moreover, disposable condoms were wrapped on the laryngoscope to reduce surgical infections.

The air management included the regular disinfection of the air conditioner cabinets in the surgical rooms (10). A well-calibrated thermostat was maintained to attain the proper temperature inside the room and prevent sweat on the face of the operators from falling into the incision accidentally. Movement of personnel was reduced as much as possible during surgery. A wet mop was used for cleaning the floor after each surgery. Others: If surgical patients had a mild upper respiratory tract infection, they wore a double layer mask during surgery. If the upper respiratory tract infection was severe, surgery participation was postponed. When selecting wound dressings, good antibacterial properties and permeability of the material were taken into consideration. Patients were given prophylactic antibacterial before and during surgery. In cases in which an operator’s skin was damaged, double layer gloves were worn. During contaminated surgeries, gauze pads were used to protect adjacent tissues, and immediate suction was applied to overflowing contents following incisions; additionally, iodophor was used for disinfection. After each contaminated operation, instruments and dressings exposed to contaminants were evacuated from the operating tables quickly; the operators wore new gloves, and closed drainage was applied wherever possible.

### Postoperative nursing management

Skin on the incision site was disinfected followed by pasting a membrane and inspecting it regularly. The membrane was changed frequently to prevent postoperative incision infection. The floor in the surgical room was cleaned and disinfected, and the surgical table drape was changed. The drapes were washed with disinfectant and then fumigated, followed by ultraviolet radiation disinfection (12). In terms of disposable surgical instruments and tools, they were destroyed after pretreatment. In addition, non-disposable surgical instruments were individually disinfected and then routinely sterilized to re-use them.

### Project report

A discussion about the project reports was held every month to analyze the ongoing trial’s partial results and analyze special cases, point out the shortcomings of the surgical room management plan and make adjustments for improvement as needed.

### Observations

The surgical room infection control was monitored by means of the colony forming units from the air, surfaces and personnel’s hands samplings. The postoperative infection rates of surgical patients in the two groups were compared. A self-made questionnaire was applied to investigate the degree of patient satisfaction with the nurses in the two groups; the opinion poll included a total of 10 questions and scored 0–1 point each, the total was rated as very satisfied (≥8), satisfied (6–7), dissatisfied (<7).

### Statistical analysis

The SPSS 22.0 software (Chicago, IL, USA) was used for data processing and statistical analysis. Measurement data were expressed as X±Sd; and comparison between groups was done by *t* test. Count data were expressed by the number of cases or a percentage; and comparison between groups was done by a *χ*^2^ test. Ranked data were tested by Mann-Whitney U method. A *P*<0.05 indicated that the differences found were statistically significant.

## Results

### Comparison of quality of measures for controlling of operation room infections between the two groups

The air, surfaces and personnel’s hands samplings in the control group yielded significantly more colony forming units than in the observation group (*P*<0.05), showing the infection control was better achieved in the observation group (Table 1).

**Table 1:** Comparison of operation room infection control measures’ quality between the two groups [n (%)]

**Group**	**Number of cases**	**Operation room air quality**	**Surface quality**	**Personnel’s hands surface quality**
Observation group	382	368 (96.34)	349 (91.36)	364 (95.29)
Control group	347	291 (83.86)	262 (75.50)	301 (86.74)
χ^2^ value		32.592	33.700	31.878
*P* value		<0.001	<0.001	<0.001

### Comparison of postoperative infection rate between the two groups

Three cases in the observation group had postoperative infections (an infectious rate of 0.79%). All the three cases were abdominal surgeries. The infections occurred 8 ∼ 70h after the operation. The infections were controlled by active antibiotic treatments. Twelve cases in the control group had postoperative infections (an infectious rate of 3.46 %). There were six cases of abdominal surgeries, 3 cases of traumatic surgeries, 2 cases of burn surgeries and one case of brain surgery. The infections occurred 10 ∼ 80h after the operation.

Nine patients were cured with antibiotic treatment, and the other 3 died unfortunately. The overall infection rate of patients in the observation group was significantly lower than that in the control group (*χ*^2^=6.446, *P*=0.011).

### Comparison of satisfaction degree of patients between groups

The degree of satisfaction of the patients in the observation group was significantly higher than that of the patients in the control group (*P*<0.05) (Table 2).

**Table 2:** Comparison of degree of satisfaction of patients in the two groups [n (%)]

**Group**	**The number of cases**	**Very satisfied**	**Satisfied**	**Dissatisfied**	**Z value**	***P* value**
Observation group	382	226 (59.16)	148 (38.74)	8 (2.09)	13.352	0.001
Control group	347	185 (53.31)	135 (38.90)	27 (7.78)		

## Discussion

The factors playing a role in the operation room infection rate include the characteristics inherent to the patients, and the operation room environmental, invasive, and managerial factors (13). The sharp reduction of body functions and organ vitality during the perioperative period results in decreased immune-competence and defense ability, so the surgical incision infection rate of the patient is significantly increased (14).

Substandard air quality in the operating room and unqualified disinfection of hands of medical personnel, surgical materials or equipment and surfaces may lead to increased infection rate of surgical patients (15). The frequent movement of medical staff, a high number of people involved and the presence of confusing management directives during an operation are all risk factors for surgical infection of the patients (16). Currently, continuous improvements have been made to the conventional OR care. Good progress has been achieved in aspects such as sterilization, equipment management, strict check, nurse job responsibilities and skills, comprehensive quality evaluation. These measures have been effective in the control of nosocomial infections and perioperative complications. However, there are still problems to be solved. Examples include the lack of standardized and specialized nursing protocols; the existence of big differences in the outcomes of operations of the same type cared by different nursing teams; the lack of sufficient study of typical cases, and the lack of individualized care for different patients who undergo the same operations. In order to decrease the risk of the operation room infection, our hospital put forward some project management measures for the first time. The project management plan was to optimize the measures for each particular project or program. Through the establishment of an operation room nursing project management team, the head nurse was able to implement an operation project for each patient with a specially assigned person to supervise and record the nursing situation before, during and after the surgery.

The results showed that the overall infection and the postoperative infection control rates of the observation group were significantly higher than that of the control group. Meanwhile, the degree of satisfaction with the nurses’ work among patients in the observation group was significantly higher than that among the patients in the control group.

These results suggested that implementing a nursing project management contributes to the improvement of measures for surgical disinfection, effectively reduces the incidence of the operation room infection and improves the surgical care satisfaction of patients. At present, the nursing project management plan has gone through continuous improvements and revisions and has formed unified and defined regulations. The applications of the nursing project management plan in our city, our province and the neighboring provinces have proved to be successful, achieving better care and clinical results.

Currently, there are few researches working on the application of the nursing project management to control the operating room infections (17, 18). There is also little experience in making the nursing project management a unified and defined protocol for the promotion of its applications. However, the nursing project management plan is effective to reduce infections in the operating room, improve the efficiency of aseptic management, optimize the quality of perioperative care and has important application values. The application of the nursing project management in the OR can facilitate the establishment of specialized care. It can also easily adapt to constantly developing surgical methods, especially different kinds of minimally invasive surgeries such as microscopic, endoscopic and interventional surgeries, as well as various high-risk surgeries such as organ transplants, artificial organs and hybrid operations. The study and discussion of typical cases on a regular basis can enhance the knowledge and skills of doctors and nurses, thus can reduce the risk of operation and avoid medical disputes to the maximum level. However, currently, there are stillrooms for improvements in the implementation of the nursing project management. Some doctors and nurses have not had a good understanding of this management plan. The enforcement is not strong enough, and there has not been an appropriate evaluation system. We propose that the integration of the nursing project management plan into the reward and performance system can significantly speed up the pace of its implementation.

The operation room, while playing an important function in the hospital, is an area of high incidence of hospital-acquired infections. Therefore, taking an effective operation room management system ensures the best measures are implemented to reduce the risk of infection to a minimum. Project management analyzes each patient’s case as an independent unit, and makes sure specific measures are in place during the preoperative, intraoperative, and postoperative periods, significantly reducing the operating room infection rate and improving the patient’s satisfaction with the nursing care and surgical outcome. Consequently, a nursing project management is worthy of clinical popularization and application. The weakness of the study is that we did not analyze the factors that influenced the nursing project management; therefore, we could not pinpoint the specific infection-controls that are improved by this management plan, making the subsequent improvement work more difficult.

## Conclusion

A nursing project management is very important for surgical patients in hospitals to popularize and apply a good nursing project management plan.

## Ethical considerations

Ethical issues (Including plagiarism, Informed Consent, misconduct, data fabrication and/or falsification, double publication and/or submission, redundancy, etc) have been completely observed by the authors.
